# Better quadriceps and hamstring strength is achieved after Total knee Arthroplasty with single radius femoral prostheses: a retrospective study based on isokinetic and isometric data

**DOI:** 10.1186/s42836-020-0022-4

**Published:** 2020-02-07

**Authors:** Mengyuan Li, Lei Zhang, Ruiying Zhang, Yuanchen Ma, Junxing Liao, Qingtian Li, Zhantao Deng, Qiujian Zheng

**Affiliations:** 1 Division of Joint Osteopathy and Traumatology, Center of Orthopedics Surgery, Guangdong Provincial People’s Hospital, Guangdong Academy of Medical Sciences, 106, Zhongshaner Road, Yuexiu District, Guangzhou, People’s Republic of China; 2 Division of Rehabilitation, Guangdong Provincial People’s Hospital, Guangdong Academy of Medical Sciences, Guangzhou, Guangdong Province People’s Republic of China

**Keywords:** Total knee arthroplasty, Single radius, Multiple radiuses, Isokinetic strength

## Abstract

**Background:**

Strength deficits, muscle imbalances, and quadriceps inhibition are common after the total knee arthroplasty (TKA). It was suggested that theoretically single radius (SR) femoral protheses could provide longer extensor moment arm compared to the multiple radius (MR) design. However, quantitative evidence has not yet been reported. Thus, the aim of the study was to investigate the differences in isokinetic data and to compare the patient-reported outcome scores between TKA SR and MR design.

**Method:**

The present retrospective study included 36 TKA involving 16 knees (9 patients) using SR design implant and 20 knees (11 patients) using MR design implant. The mean follow-up time was longer than 1 year. Isokinetic knee flexion and extension torques of the operated leg were evaluated at 60°/s and 180°/s. Quadriceps and hamstring torques and ratios, work and power were recorded. Angle-specific torques were also collected at different extension or flexion angles.

**Results:**

Both groups showed improvement in knee society scores (KSS) and knee injury, and osteoarthritis outcome score (KOOS) after operation. Patients in SR group had significantly higher scores in KSS-knee, symptoms and activities of daily living KOOS sub-score than those in the MR group at the end of the follow-up. The peak knee flexion torque, peak knee extension torque and maximum knee flexion work were greater in SR group at 180°/s and 60°/s. At 60°/s, and SR group had higher average knee flexion power and average knee extension power than MR group. In the isometric contraction test, the knee extension torque was higher in SR group than in MR group. At 180°/s, SR group showed higher flexion torques at 30°, 40°, 50°, 60° compared with MR group. At 60°/s, SR group showed higher flexion torques at 30°, 40°, 50°, 60°, 80° when compared with MR group. Additionally, SR group also provided higher extension torques at 40°, 50°, 60° than the MR group. There were no differences in other isokinetic and isometric parameters between the two groups.

**Conclusion:**

Femoral design exerted an influence on quadriceps and hamstring strength after TKA, and SR design shows advantages, in terms of higher extension and flexion strength, over MR design.

## Introduction

Total knee arthroplasty (TKA) is highly effective in reducing pain and enhancing function in those suffering from advanced osteoarthritis and rheumatoid arthritis [1–4]. Adequate function of the extensor mechanism after TKA is essential for a satisfactory clinical outcome and for daily living activities [5]. Although most patients do well, some report a less satisfactory outcome and moderate rates of dissatisfaction are consistently reported in around 20% of patients [6]. The possible reasons included anterior knee pain, instability, limited range of motion, and extensor insufficiency, which are probably related to the kinematics of the knee prostheses [1–4, 7]. Single radius (SR) and multi-radius (MR) femoral designs are believed to impact, to different degrees, on the biomechanism of the knee [8, 9]. First of all, with SR strategy, the femoral-tibial contact point is more posterior, and, hence, the SR implant improves the mechanical efficiency by providing a longer extensor moment arm and reducing the pressure on the patellofemoral joint [10, 11]. Secondly, the SR configuration maintains the collateral ligaments in an isometric form during knee movement, thereby providing sustained stability. Conversely, prostheses with MR design lead to mid-flexion instability and femoral paradoxical anterior movement because of the laxity of the collateral ligaments due to the change in condylar radius [12, 13].

Clinical studies that compared the SR and MR femoral design yielded contradictory results [14–19]. Liu *et al*. conducted a meta-analysis in order to find the difference in clinical outcomes between the SR and MR femoral design. However, it failed to show any theoretical advantages of SR design over its MR counterpart [20]. Some studies compared the strength of lower limb between SR and MR TKA, but these qualitative researches used simple physical examinations, such as sit-to-stand test, or measured the strength on a static basis [14, 18], and the results were not conclusive.

Isokinetic analysis can measure the kinetic parameters of muscles at a specific velocity, which allows the comprehensive evaluation of function of the muscles around the joint. Up till now, few comparative trials reported the isokinetic characteristics of SR and MR designs [21]. The purpose of the current study was to investigate the differences in isokinetic data and to compare the patient-reported outcomes between TKA SR and MR design.

Based on the theoretical advantage of the SR design documented in literature, we came up with the following hypotheses: (1) the patient-reported outcomes, in terms of Knee Society Scores (KSS) and Knee Injury, and Osteoarthritis Outcome Score (KOOS), differ between the SR and MR knees; (2) the isokinetic torques, hamstring/quadriceps (H/Q)-ratios, isokinetic work and power are different between the SR and MR knees, and, furthermore, these differences vary with the knee flexion angle and movement velocity.

## Materials and methods

This retrospective, comparative study was approved by the ethics committee of our medical university and was performed in strict accordance with the ethical standards stipulated in the 1964 Declaration of Helsinki and its later amendments. Written informed consent was obtained from all patients before enrollment.

Inclusion criteria were as follows: The subjects (1) were diagnosed with primary symptomatic osteoarthritis of the knee; (2) had a nonfixed varus or valgus deformity of < 10°; (3) were 55 to 85 years old; (4) had a body mass index (BMI) lower than 35 kg/m^2^; (5) satisfied the criteria for class 1 or 2 of the American Society of Anesthesiologists (ASA); and (6) had provided informed consent. Exclusion criteria included: (1) suffering from inflammatory arthritis, including rheumatoid arthritis, suppurative arthritis and gouty arthritis; (2) having previous history of unicondylar or total knee replacement; (3) having past history of tibial/femoral osteotomy; (4) flexion < 90°; (5) flexion contracture/extension deficit > 10°; (6) varus / valgus malalignment > 10°; or (7) concomitantly having any other lower extremity diseases.

Surgery was performed by the same group of surgeons (QJZ, YZM, JXL). All surgical procedures were done under general or spinal anesthesia under tourniquet control and after standard antibiotic prophylaxis. The knee was exposed with a straight anterior skin incision and a straight medial parapatellar capsular incision was made for the arthrotomy. The patella was everted and was not resurfaced in all cases. Posterior cruciate ligament (PCL)-substituting design was applied. The standard instrumentation was employed, and measured resection technique was utilized to achieve appropriate component alignment. The Stryker Triathlon TKA system (Stryker Orthopaedics, Mahwah, New Jersey) was used in SR group and either PFC sigma (DePuy Orthopaedics, Inc. Warsaw, IN, USA) was used in MR group. All patients were put on the same standardized rehabilitation program. Patients were mobilized from the second postoperative day under supervision of our physiotherapists. Exercises included continuous passive motion, assisted and unassisted knee extension, walking and stair climbing with 2 crutches, and progression as tolerated.

**Fig. 1:**
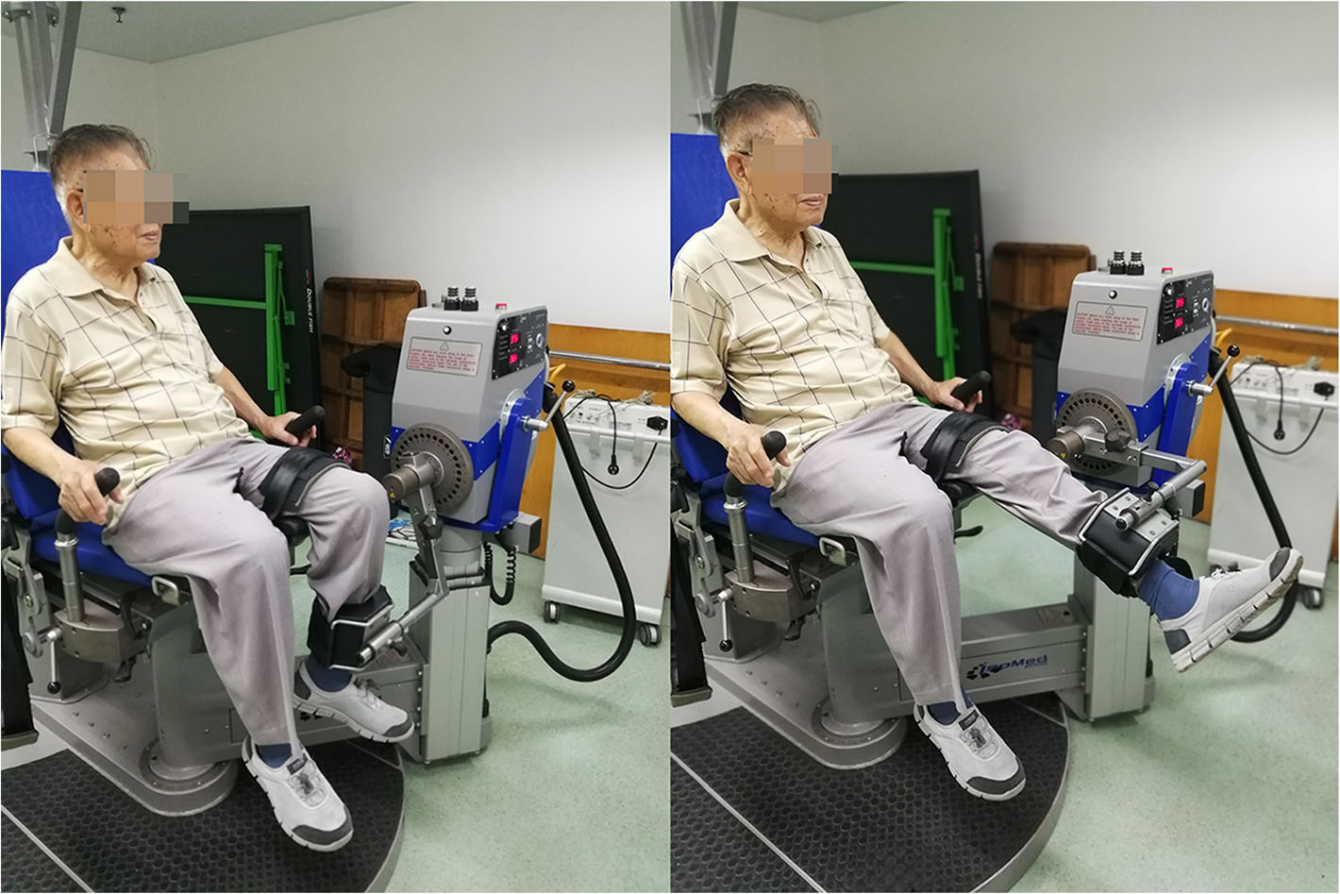
Isokinetic torque measurement with the IsoMed 2000 dynamometer

The flexion and extension isokinetic strengths of knees were measured in the seated position at an angular velocity of 180°/s using an isokinetic dynamometer (IsoMed 2000, D&R GmbH, Hemau, Germany) (Fig. 1). Six duplicate leg extension and flexion measurements from a resting knee joint angle of 10° were made with adequate rest periods in between efforts. After a short period of recovery, the muscle endurance of the knee extensors was obtained by measuring the total work achieved during another series of six consecutive repetitions, at an angular velocity of 60°/s. Finally, knee flexion and extension isometric torques were recorded. The work, maximum extension and flexion torques, average extension and flexion power, and the extension and flexion torques at every 10° from 10° to 80° were recorded for further analysis. Finally, the isometric extension and flexion torques were also measured. KSS and KOOS scores were collected pre- and post-operatively.

The SPSS 22.0 software package (IBM Inc. USA) was used for statistical analysis. Fisher’s exact test was used for the categorical variables. Paired-samples T test was used to compare the KSS, KOOS scores and isokinetic parameters pre- and post-operatively. The subjective scores and isokinetic parameters between SR and MR group were compared using One-way ANOVA. A post-hoc power calculation was determined by the statistical power analyses (G Power 3.1) to eliminate type II error. A level of *P* < 0.05 was set for statistical significance.

## Results

A total of 20 patients were included in this study, including 9 (16 knees) and 11 patients (20 knees) in SR and MR group, respectively. There were no differences between the two groups in demographic data including sex, age, height, weight and body mass index, and distribution in receiving bilateral/unilateral TKA (*P* > 0.05). The data of the two groups were homogeneous (Table 1). Post-hoc power analysis showed a power > 0.76 for detecting a significant difference.

**Table 1:** Demographic and clinical data (mean ± SD) of included patients

	SR group (*n* = 9)	MR group (*n* = 11)	*P* value
Sex, male:female, n	3/6	4/7	1.000
Age, y	62.6 ± 4.8	64.7 ± 5.3	0.853
Height, m	1.66 ± 0.09	1.55 ± 0.06	0.159
Weight, kg	71.44 ± 13.13	63.64 ± 16.31	0.261
Body mass index	23.55 ± 6.93	26.31 ± 5.72	0.702
Unilateral/Bilateral TKA, n	2/7	2/9	1.000

*SD* standard deviation, *SR* single radius, *MR* multiple radius, *TKA* total knee arthroplasty

Both groups showed an improvement in KSS and KOOS score post-operatively when compared with the pre-operative level. The SR group scored significantly better for the KSS-knee, Symptom and Activities of daily living KOOS sub-score post-operatively (*P* < 0.05 for each). There existed no significant differences in the clinical outcome scores between the two groups in other measures (Table 2).

**Table 2:** KSS and KOOS Scores (mean ± SD) before and after TKA

	Preoperative			Postoperative		
	MR group	SR group	*P* value	MR group	SR group	*P* value
KSS-Knee	49.8 ± 19.3	53.3 ± 14.78	0.524	71.1 ± 20.6	83.9 ± 13.6	0.049
KSS-Function	51.8 ± 15.0	56.2 ± 20.6	0.538	75.5 ± 21.8	82.3 ± 26.3	0.545
KOOS-Pain	51.5 ± 10.1	48.6 ± 16.9	0.756	74.6 ± 14.4	76.5 ± 19.3	0.746
KOOS-Symptom	53.6 ± 14.7	49.7 ± 9.67	0.673	72.1 ± 22.1	86.7 ± 16.0	0.043
KOOS-Activities of daily living	50.6 ± 17.2	46.5 ± 16.11	0.746	70.1 ± 18.1	87.2 ± 15.8	0.008
KOOS-Sports/recreation	10.5 ± 7.6	12.3 ± 8.4	0.874	57.5 ± 25.1	62.1 ± 33.0	0.551
KOOS-Quality of life	24.7 ± 13.0	24.5 ± 14.3	0.995	57.5 ± 19.7	66.5 ± 21.2	0.212

*KSS* Knee Society Scores, *KOOS* Knee Injury, and Osteoarthritis Outcome Score, *SD* standard deviation, *TKA* total knee arthroplasty, *SR* single radius, *MR* multiple radius

There were no statistical differences between the SR and MR group in peak knee flexion torque, peak knee extension torque, H/Q ratio, maximum knee flexion work, maximum knee extension work, average knee flexion power, average knee extension power respectively at the velocity of either 180°/s or 60°/s (*P* > 0.05 for all) before TKA. After TKA, all the parameters improved compared with the figures before TKA. When comparing the SR and MR group, the peak knee flexion torque and peak knee extension torque were higher in SR group at 180°/s and 60°/s (*P* > 0.05 for both velocity). SR group showed higher maximum knee flexion work at either velocity (*P* < 0.05). At 60°/s, SR group had higher average knee flexion power and average knee extension power than MR group. Additionally, in the isometric contraction test, the knee extension torque was higher in SR group than in MR group (Table 3).

**Table 3:** Isokinetic and isometric parameters (mean ± SD) between groups before and after TKA

velocity	parameters	Preoperative			Postoperative		
		MR group	SR group	*P* value	MR group	SR group	*P* value
180°/s	Peak knee flexion torque (N.m)	17.45 ± 8.35	19.13 ± 5.67	0.498	25.25 ± 9.97	39.50 ± 19.20	0.014
	Peak knee extension torque (N.m)	23.60 ± 15.95	26.94 ± 8.34	0.454	38.05 ± 17.07	54.88 ± 28.29	0.034
	H/Q ratio	0.81 ± 0.20	0.73 ± 0.16	0.177	0.77 ± 0.42	0.74 ± 0.15	0.809
	Maximum knee flexion work (J)	15.30 ± 3.16	17.44 ± 7.46	0.018	19.95 ± 8.61	30.75 ± 14.72	0.016
	Maximum knee extension work (J)	19.60 ± 6.83	23.63 ± 8.63	0.127	36.80 ± 19.33	50.25 ± 26.66	0.088
	Average knee flexion power (W)	20.75 ± 10.04	26.63 ± 12.18	0.122	28.95 ± 17.60	44.38 ± 33.07	0.106
	Average knee extension power (W)	32.45 ± 11.14	35.88 ± 13.15	0.403	55.40 ± 31.96	70.13 ± 52.31	0.305
60°/s	Peak knee flexion torque (N.m)	32.90 ± 9.61	36.31 ± 11.61	0.802	32.70 ± 11.04	49.3 ± 23.66	0.017
	Peak knee extension torque (N.m)	47.40 ± 21.38	59.69 ± 19.66	0.085	52.75 ± 25.05	76.31 ± 34.96	0.032
	H/Q ratio	0.60 ± 0.25	0.63 ± 0.34	0.689	0.76 ± 0.46	0.67 ± 0.17	0.421
	Maximum knee flexion work (J)	34.95 ± 8.99	34.19 ± 10.93	0.468	28.15 ± 10.01	42.38 ± 16.55	0.006
	Maximum knee extension work (J)	46.45 ± 21.85	60.38 ± 18.95	0.052	52.05 ± 28.48	70.50 ± 28.89	0.063
	Average knee flexion power (W)	15.35 ± 5.89	17.56 ± 6.02	0.275	18.75 ± 8.68	36.31 ± 25.85	0.018
	Average knee extension power (W)	31.70 ± 5.68	33.19 ± 9.79	0.728	35.15 ± 24.86	60.81 ± 42.46	0.043
Isometric contraction	Knee flexion torque (N.m)	36.75 ± 12.67	40.81 ± 12.80	0.348	51.00 ± 19.74	70.38 ± 37.76	0.077
	Knee extension torque (N.m)	49.00 ± 18.95	52.62 ± 18.07	0.036	57.90 ± 28.69	94.69 ± 46.38	0.011

*SD* standard deviation, *TKA* total knee arthroplasty, *SR* single radius, *MR* multiple radius, *H/Q* hamstring/Quadriceps

**Fig. 2:**
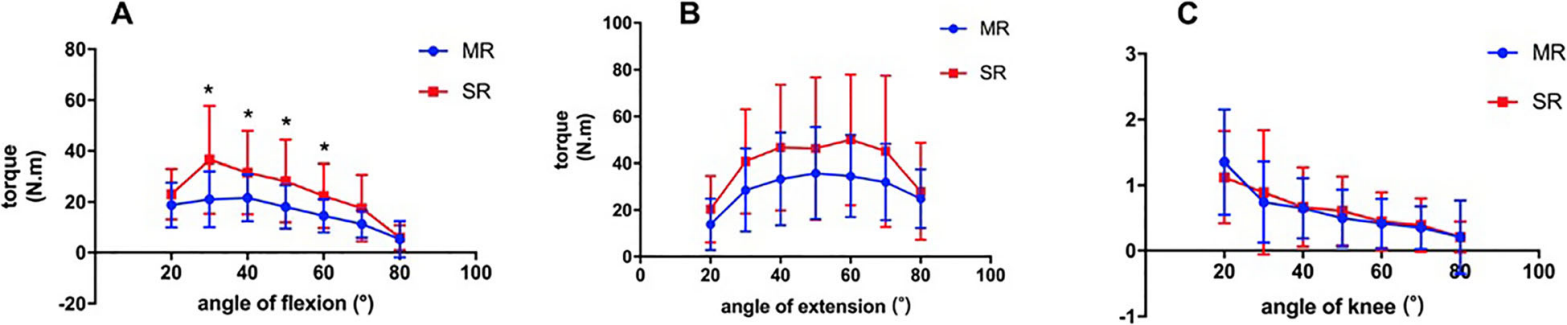
Comparison of angle specific torque at 180°/s: **a** extension; **b** flexion; **c** H/Q ratio. * indicated *P* < 0.05 between groups. (SR: single radius, MR: multiple radius, H/Q: hamstring/quadriceps)

The angle-specific extension, flexion torque values and H/Q ratio are shown in Tables 4, 5, 6 and Fig. 2, 3. For both velocities in knee flexion, significantly higher torque could be seen at 30°, 40°, 50°, and 60° in SR group (*P* < 0.05), and, moreover, the flexion torque was higher in SR group at 80°, 60°/s (*P* < 0.05). During extension, the torque value was higher at 40°, 50°, and 60° in SR group only at 60°/s (*P* < 0.05). Although the extension torque value was higher in SR group at 180°/s, the differences were not statistically significant (*P* > 0.05). There were no differences in H/Q ratio during the range of motion (ROM) in both groups at both velocities.

**Table 4:** Angle-specific extension torque (mean ± SD) at 180°/s and 60°/s

velocity	angle	MR group	SR group	*P* value
180°/s	20°	13.85 ± 11.14	20.38 ± 14.26	0.132
	30°	28.55 ± 17.76	40.81 ± 22.25	0.075
	40°	33.25 ± 19.85	46.69 ± 26.86	0.093
	50°	35.80 ± 19.70	46.31 ± 30.53	0.244
	60°	34.50 ± 17.52	50.06 ± 27.94	0.064
	70°	31.95 ± 16.29	45.13 ± 32.30	0.152
	80°	24.90 ± 12.62	27.94 ± 20.79	0.592
60°/s	20°	29.10 ± 17.31	35.00 ± 18.57	0.332
	30°	41.50 ± 22.86	55.69 ± 23.06	0.074
	40°	45.25 ± 25.73	67.50 ± 29.19	0.021
	50°	45.10 ± 28.83	71.63 ± 33.00	0.015
	60°	47.70 ± 26.45	71.56 ± 34.46	0.025
	70°	42.80 ± 27.66	59.63 ± 36.41	0.124
	80°	35.79 ± 20.31	31.50 ± 25.60	0.584

*SD* standard deviation, *SR* single radius, *MR* multiple radius

**Table 5:** Angle-specific flexion torque (mean ± SD) at 180°/s and 60°/s

velocity	angle	MR group	SR group	*P* value
180°/s	20°	18.75 ± 8.84	23.00 ± 9.95	0.184
	30°	21.00 ± 11.03	36.50 ± 21.25	0.015
	40°	21.65 ± 9.24	31.50 ± 16.40	0.043
	50°	18.05 ± 8.58	28.19 ± 16.29	0.035
	60°	14.50 ± 6.66	22.38 ± 12.64	0.035
	70°	11.25 ± 5.47	17.56 ± 13.14	0.087
	80°	5.25 ± 7.17	5.88 ± 4.92	0.769
60°/s	20°	28.00 ± 10.91	30.44 ± 15.78	0.588
	30°	31.60 ± 11.21	46.25 ± 23.88	0.035
	40°	29.05 ± 10.63	46.06 ± 24.00	0.016
	50°	25.00 ± 9.28	42.94 ± 20.99	0.005
	60°	20.70 ± 8.12	30.75 ± 11.96	0.005
	70°	14.95 ± 7.08	19.31 ± 14.46	0.282
	80°	7.21 ± 6.71	15.36 ± 12.30	0.037

*SR* single radius, *MR* multiple radius

**Table 6:** Angle-specific H/Q ratio (mean ± SD) at 180°/s and 60°/s

velocity	angle	MR group	SR group	*P* value
180°/s	20°	1.35 ± 0.80	1.12 ± 0.70	0.976
	30°	0.74 ± 0.62	0.89 ± 0.95	0.834
	40°	0.65 ± 0.46	0.67 ± 0.61	0.899
	50°	0.50 ± 0.44	0.61 ± 0.53	0.782
	60°	0.42 ± 0.38	0.45 ± 0.45	0.816
	70°	0.35 ± 0.33	0.39 ± 0.41	0.917
	80°	0.21 ± 0.56	0.21 ± 0.24	0.798
60°/s	20°	0.96 ± 0.63	0.87 ± 0.85	0.838
	30°	0.76 ± 0.49	0.83 ± 1.03	0.830
	40°	0.66 ± 0.41	0.68 ± 0.48	0.791
	50°	0.55 ± 0.32	0.60 ± 0.64	0.886
	60°	0.43 ± 0.31	0.43 ± 0.35	0.893
	70°	0.35 ± 0.26	0.32 ± 0.40	0.758
	80°	0.20 ± 0.33	0.49 ± 0.48	0.095

*SR* single radius, *MR* multiple radius, *H/Q* hamstring/quadriceps

**Fig. 3:**
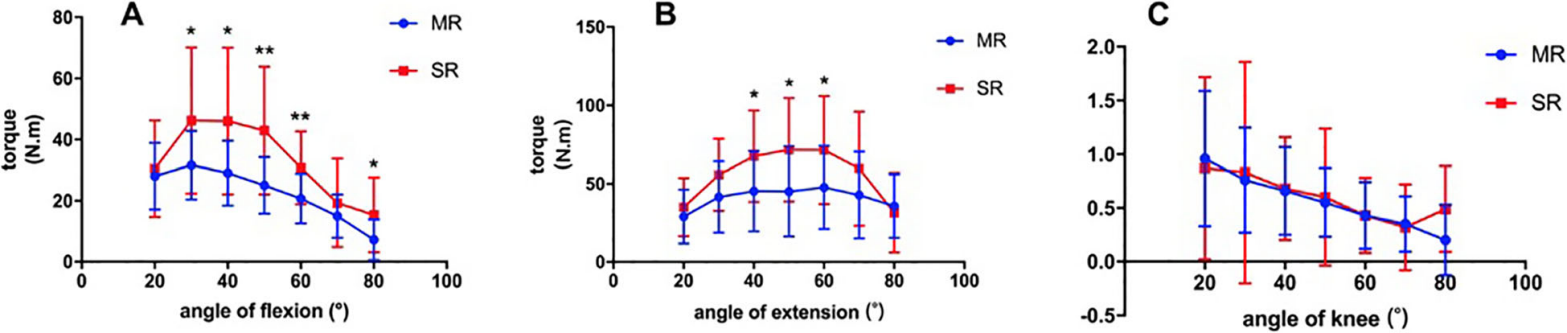
Comparison of angle specific torque at 60°/s: **a** extension; **b** flexion; **c** H/Q ratio. * indicated *P* < 0.05 between groups and ** indicated *P* < 0.01 between groups. (SR: single radius, MR: multiple radius, H/Q: hamstring/Quadriceps)

## Discussion

Strength deficits, muscle imbalances, and quadriceps inhibition are common after the total knee arthroplasty (TKA). It was suggested that theoretically single radius (SR) femoral protheses could provide longer extensor moment arm and maintain the collateral ligaments in an isometric form during knee movement, which could improve the outcome of TKA. Our current retrospective study was designed to investigate the potential theoretical advantages of SR femoral design. In addition to better clinical scores in SR groups, the main finding was that femoral design affects both Quadriceps and Hamstring strength after TKA, and SR design showed higher extension and flexion strength, especially in the middle of ROM.

Currently, some large-sized trials, irrespective of retrospective or randomized design, showed positive results in favor of the SR design. Cook *et al*. [15] compared 426 cases of SR and 113 cases of MR designs in a follow-up lasting 3.9-years on average and reported that the SR group had a significantly better KSSs, flexion, stability, pain, gait, and stair climbing. Palmer *et al*. [22] compared 388 cases of SR and 674 cases of MR, and they found a significantly better flexion and KSSs in the SR group upon either 1- or 2-year follow-up than in the MR group, and 66.3% of their patients didn’t experience any pain after 2 years against 54.4% of the patients with MR knees. Collados-Maestre* et al*. [18] conducted the largest-sized RCT comparing the clinical outcomes and reported significant better KSSs, ROM, extension lag, quadriceps strength, chair test, and WOMAC pain and a higher satisfaction rate in the SR group. In our study, there were no significant differences in KSS-knee, function score and all the aspects of KOOS score, which is coincident with the previously reported findings [15, 18, 22].

The SR implant works better since it can optimize the extensor’s function. D’Lima *et al*. [23] reported approximately 1 cm posterior of the femorotibial contact point in SR design as compared to the MR design, which can lower the quadriceps and patellofemoral forces that are required in the knee extension. In a cadaveric study, the SR knee required 57% less quadriceps force as compared to the MR knee, and the author inferred that SR design could reserve the strength of the extensor mechanism substantially [24]. Collados-Maestre *et al*. [18] reported that the SR group showed a significantly better quadriceps strength than the MR group. Mahoney *et al*. [8] found that 90% of the patients in the SR group, which was significantly higher than the proportion in the MR group, could rise from a folded chair independently at 2 years. Wang *et al*. [12] reported that a prolonged duration was required in patients undergoing the MR design to perform the sit-to-stand test than those undergoing the SR design as assessed by 3-dimensional kinematics. Larsen *et al*. [17] and Kim *et al*. [19] reported higher extensor strength in SR knees than that in their MR counterparts. Although SR protheses showed better results in the current literature, isokinetic strength test was not often done in the context of SR or MR TKA. Gómez-Barrena *et al*. [21] conducted an isokinetic study and observed better extensor performance with decreased flexion peak torque and increased extension peak torque in patients with SR design than those with MR design. In the present study, the isokinetic parameters of knee extensor were higher in SR group that in MR group, which was consistent with the results of previous study. Therefore, the SR design may be advantageous to the extensor mechanism function.

The angle-specific analysis of isokinetic torque data provided more details than conventional data analyses based on single parameters. However, the characteristics of angle-specific isokinetic torque have not yet been reported in the current literature. In our study, we found that in the middle of ROM, the angle-specific torques were higher in SR group than in MR group. Additionally, the angle-specific torque curves of the SR and MR group had different shapes. In the curves of SR group, the latitude of change in extension and flexion torque was more significant. On the contrary, the change of torque was smaller in the MR group at both 60°/s and 180°/s. Lower strength in the middle of ROM in MR group might be indicative of slight instability and femoral paradoxical anterior movement due to the change in condylar radius in the MR design during motion [25]. Hamstrings and capsule contraction, to a significant extent, to compensate for the laxity of knee, and excessive mechanical stresses on the soft tissues stimulate fibrous hyperplasia and knee joint synovitis with consequent knee stiffness. We inferred that part of extensor mechanism fibers might be recruited to compensate for this phenomenon, leading to loss in actual extension strength. Therefore, the SR protheses do provide better quadriceps functions than MR design. Besides, it is worth noticing that although the SR group showed better average knee extension power, mid-ROM torques and isometric knee extension torque only at 60°/s, it failed to yield the similar results at 180°/s. Thus, SR knee showed better endurance during the motion of knee. Nonetheless, the ability to produce high-speed knee movement and the explosive power of knee was impaired to some extent in TKA knees regardless of prothesis designs.

Of note, the difference in the flexor torque in our study might be conflicting. The decreased flexion torque is a frequent finding in TKA isokinetic studies [26]. However, our study exhibited that the peak flexor parameters and mid-ROM flexion torques were higher in scale with extensor figures in SR group, and thus there was no substantial difference in the H/Q ratio. This last parameter best describes the recovery of the muscular function and has been proven to fall below normal values in TKA patients with different designs even after long-term follow-up [27], with a normal value of 0.5–0.8 [28]. A more favorable H/Q ratio depends not only on the increase of extensors but also on the decrease of flexors [21]. These results implied that although the SR group showed better recovery in extensor mechanism, it did not recovered to the ideal level. That might be attributed to persistent muscle weakness, surgical trauma during TKA, and age-related muscle recovery dysfunction. Thus, minimally invasive surgery (MIS) and persistent rehabilitation may be beneficial for longer-term recovery.

This study had some limitations. First, the sample size was small and the follow-up time points were not consistent. Despite this, the statistical analysis indicated the results had reliable reproducibility. A larger-sized, multi-center, long-term study is warranted in future. Second, there was no specific post-operative rehabilitation protocol for the patients. Third, we only collected the isokinetic data as objective results, and it would be interesting to analyze the gait characteristics to see further subtle differences between the groups. Fourth, the potential similar performance of bilateral knees in the same patient might decrease the validating power. Last but not least, the study failed to randomize the surgical groups, which made the patient selection a confounder.

## Conclusion

Femoral design exerted an influence on quadriceps and hamstring strength after TKA. SR design showed advantages, in terms of higher extension and flexion strength, over MR design, especially in the middle of ROM.

## Data Availability

The datasets used and/or analyzed during the current study are available from the corresponding author on reasonable request.

## Abbreviations

ASA: American Society of Anesthesiologists
BMI: Body mass index
H/Q ratio: Hamstring/quadriceps ratio
KOOS: Knee Injury and Osteoarthritis Outcome Score
KSS: Knee Society scores
MR: Multi-radius
PCL: Posterior cruciate ligament
ROM: Range of motion
SR: Single radius
TKA: Total knee arthroplasty

## References

[1] National Joint Registry for England WaNI (2017). 14th Annual Report 2017.

[2] Register SKA (2017). Annual report.

[3] Registry AJR (2017). Fourth AJRR annual report on hip and knee arthroplasty data.

[4] Registry AOANJR (2017). Hip, knee and shoulder arthroplasty annual report.

[5] Andriacchi TP, Yoder D, Conley A (1997). Patellofemoral design influences function following total knee arthroplasty. J Arthroplast.

[6] Carr AJ, Robertsson O, Graves S (2012). Knee replacement. Lancet..

[7] Frankel VH, Burstein AH, Brooks DB (1971). Biomechanics of internal derangement of the knee. Pathomechanics as determined by analysis of the instant centers of motion. J Bone Joint Surg Am.

[8] Mahoney OM, McClung CD (2002). dela Rosa MA, et al. the effect of total knee arthroplasty design on extensor mechanism function. J Arthroplast.

[9] Kessler O, Durselen L, Banks S (2007). Sagittal curvature of total knee replacements predicts in vivo kinematics. Clin Biomech (Bristol, Avon).

[10] Jenny JY, Miehlke R, Saragaglia D (2013). Single-radius, multidirectional total knee replacement. Knee Surg Sports Traumatol.

[11] Ezechieli M, Dietzek J, Becher C (2012). The influence of a single-radius-design on the knee stability. Technol Health Care.

[12] Wang H, Simpson KJ, Ferrara MS (2006). Biomechanical differences exhibited during sit-to-stand between total knee arthroplasty designs of varying radii. J Arthroplast.

[13] Stoddard JE, Deehan DJ, Bull AM (2013). The kinematics and stability of single-radius versus multi-radius femoral components related to mid-range instability after TKA. J Orthopaedic Res.

[14] Hall J, Copp SN, Adelson WS (2008). Extensor mechanism function in single-radius vs multiradius femoral components for total knee arthroplasty. J Arthroplast.

[15] Cook LE, Klika AK, Szubski CR (2012). Functional outcomes used to compare single radius and multiradius of curvature designs in total knee arthroplasty. J Knee Surg.

[16] Hamilton DF, Burnett R, Patton JT (2015). Implant design influences patient outcome after total knee arthroplasty: a prospective double-blind randomised controlled trial. Bone Joint J.

[17] Larsen B, Jacofsky MC, Jacofsky DJ (2015). Quantitative, comparative assessment of gait between single-radius and multi-radius Total knee Arthroplasty designs. J Arthroplast.

[18] Collados-Maestre I, Lizaur-Utrilla A, Gonzalez-Navarro B (2017). Better functional outcome after single-radius TKA compared with multi-radius TKA. Knee Surg Sports Traumatol Arthroscopy.

[19] Kim DH, Kim DK, Lee SH (2015). Is single-radius design better for quadriceps recovery in Total knee Arthroplasty?. Knee Surg Relat Res.

[20] Liu S, Long H, Zhang Y (2016). Meta-analysis of outcomes of a single-radius versus multi-radius femoral Design in Total Knee Arthroplasty. J Arthroplast.

[21] Gomez-Barrena E, Fernandez-Garcia C, Fernandez-Bravo A (2010). Functional performance with a single-radius femoral design total knee arthroplasty. Clin Orthop Relat Res.

[22] Palmer J, Sloan K, Clark G (2014). Functional outcomes comparing triathlon versus Duracon total knee arthroplasty: does the triathlon outperform its predecessor?. Int Orthop.

[23] D'Lima DD, Poole C, Chadha H (2001). Quadriceps moment arm and quadriceps forces after total knee arthroplasty. Clin Orthop Relat Res.

[24] Ostermeier S, Stukenborg-Colsman C (2011). Quadriceps force after TKA with femoral single radius. Acta Orthop.

[25] Mugnai R, Digennaro V, Ensini A (2014). Can TKA design affect the clinical outcome? Comparison between two guided-motion systems. Knee Surg Sports Traumatol Arthroscopy..

[26] Fuchs S, Tibesku CO, Floren M (2000). Interdependence of clinical and isokinetic results after bicondylar knee prostheses with special emphasis on quality of life results. Int Orthop.

[27] Huang CH, Cheng CK, Lee YT (1996). Muscle strength after successful total knee replacement: a 6- to 13-year followup. Clin Orthop Relat Res.

[28] Kannus P, Jarvinen M (1990). Knee flexor/extensor strength ratio in follow-up of acute knee distortion injuries. Arch Phys Med Rehabil.

